# Toxicological profiling of clinically used chemotherapeutics in zebrafish *(Danio rerio)* larvae

**DOI:** 10.25122/jml-2025-0182

**Published:** 2026-05

**Authors:** Andrei Nicolae Ceobanu, Alexandru Florin Braniște, Gabriel Mihail Dimofte

**Affiliations:** 1 Department of Oncology, Grigore T Popa University of Medicine and Pharmacy, Regional Institute of Oncology, Iasi, Romania; 2 Department of Endocrinology, Grigore T Popa University of Medicine and Pharmacy, Sf. Spiridon Emergency Clinical County Hospital, Iasi, Romania; 3 Department of Surgery, Grigore T Popa University of Medicine and Pharmacy, Regional Institute of Oncology, Iasi, Romania

**Keywords:** zebrafish, chemotherapy toxicity, drug screening, personalized oncology, 5-FU, 5-fluorouracil, AAE, any adverse effects (mortality and morphological abnormalities), AC, doxorubicin and cyclophosphamide, FOLFIRI, 5-fluorouracil, folinic acid, and irinotecan, FOLFIRINOX, 5-fluorouracil, folinic acid, irinotecan, and oxaliplatin, FOLFOX, 5-fluorouracil, folinic acid, and oxaliplatin, hpf, hours post-fertilization, TU, Tübingen (wild-type zebrafish strain), CSP, Casper strain, ZF, zebrafish, zPDX, zebrafish patient-derived xenograft

## Abstract

Zebrafish patient-derived xenografts (zPDX) are a powerful emerging platform for personalized oncology, offering a rapid in vivo system for high-throughput chemoprofiling. Recent studies have demonstrated a strong predictive correlation between drug response in zPDX and patient clinical outcomes. However, current protocols show significant variability in drug concentrations, which can hinder comparison across studies. We aimed to establish toxicity profiles for commonly used clinical chemotherapeutic agents in wild-type Tübingen (TU) and Casper (CSP) zebrafish (ZF) strains. Embryos aged 48-72 hours post-fertilization (hpf) were exposed to a clinically relevant chemotherapeutic panel, including standard combination regimens, for 72 hours. Toxicity was assessed using two parameters: mortality rate and any adverse effects (AAE), defined as embryos exhibiting either mortality or morphological abnormalities. Screening of single agents was similar between the two strains, but the combination regimes revealed toxicity disparities, with AAE proving to be the more sensitive endpoint. Highly toxic agents, such as paclitaxel, caused rapid, dose-dependent lethality, whereas antimetabolites like 5-fluorouracil (5-FU) showed high safety margins. Multi-agent protocols demonstrated synergistic toxicity, with more complex regimens correlating with increased adverse effects, particularly in the CSP strain. This study establishes a toxicological framework for standardizing chemotherapy dosing in ZF larvae and recommends AAE as the primary metric for defining non-toxic concentrations. Our results also underscore the necessity of testing the exact clinical drug formulation, due to potential excipient effects, and of screening multi-agent protocols for synergistic toxicity. We hope these findings will contribute to the further standardization of zPDX models for clinical applications.

## Introduction

Zebrafish (ZF) embryos are gaining prominence in personalized oncology due to their practical advantages, including ease of manipulation, low cost [1], reduced ethical burden compared with murine models [2], high genetic homology to humans [3], functional metabolism [4], lack of an adaptive immune system in the first weeks of life [5] and the availability of diverse transgenic lines [6]. One of their primary applications is xenografting, which involves transplanting tumor-derived cells from standardized cell lines or patient-derived samples into ZF embryos for chemotherapy sensitivity assays. Many such models have been established for a variety of cancers including but not limited to colorectal [7], breast [8], ovarian [9], and lung [10]. While clinical dosages of chemotherapeutic agents are relatively standardized across treatment protocols, cancer types, and cycle durations, the concentrations employed in ZF models vary considerably between studies and authors. Currently, no universally accepted or standardized dosing regimen exists. Researchers gravitate toward selecting the highest non-toxic concentration for the embryo to maximize the chance of inducing a tumor response. Even for the same cancer type, different studies often use varying concentrations of the same chemotherapeutic agent, highlighting the lack of standardization in ZF xenograft protocols [9,11]. Moreover, significant inter-strain variability in response to toxins exists, even across different wild-type backgrounds, necessitating direct testing of the specific genotype used to ensure experimental accuracy [12].

In this study, we aimed to evaluate the toxicity of wild-type Tübingen (TU) ZF larvae to a selection of commonly used clinical chemotherapeutic agents. The drugs were sourced from the hospital pharmacy to ensure the exact formulation used in a clinical setting, as excipients and formulation additives may influence toxicity in ZF models. Following the screening in the TU strain, we selected single agents and multidrug combinations for secondary evaluation in the Casper genotype to assess whether genetic background influences the observed toxicity profiles. Doses were selected based on any adverse effects (AAE) observed in previous TU strain experiments.

## Material and methods

### Study design and setting

This was a prospective, experimental toxicology study conducted at the Regional Institute of Oncology and Alexandru Ioan Cuza University, Iasi, Romania. The study aimed to evaluate the toxicity of clinically relevant chemotherapeutics on a wild-type ZF model.

### Adult ZF housing and embryo collection

Wild-type TU adult and Casper (*mitfa* ^-/-^; *roy* ^-/-^) ZF were obtained from Alexandru Ioan Cuza University, Iasi. Fish were maintained under standard laboratory conditions at a density of 7–8 adults per liter of water, at 28–29°C, with a 14-hour light/10-hour dark cycle, and were fed twice daily. Embryos were collected in the morning after spawning into Petri dishes containing E3 medium [5 mmol/L NaCl, 0.17 mmol/L KCl, 0.33 mmol/L CaCl_2_, 0.33 mmol/L MgSO_4_, and 100 µg/mL methylene blue (1%)], with pH adjusted to 7.2.

### Inclusion and exclusion criteria

In this study, we included fertilized embryos between 48-72 hours post-fertilization (hpf) with normal morphological development. All embryos exhibiting phenotypic abnormalities, lack of fertilization, or mortality were excluded.

### Embryo dechorionation

Unhatched embryos were dechorionated using pronase (PRON-RO, Roche) at a concentration of 1 g/L. Embryos were incubated for 15 minutes in the enzyme solution and gently pipetted every few minutes using a Pasteur pipette to facilitate chorion detachment, after which they were rinsed thoroughly in fresh E3 medium and transferred to clean containers.

### Chemotherapeutics administration

A panel of clinically relevant chemotherapeutic agents was obtained from the pharmacy of the Regional Institute of Oncology, Iasi. The compounds included doxorubicin (Accord Healthcare), cyclophosphamide (Teva Pharmaceuticals), paclitaxel (Accord Healthcare), 5-FU (Ebewe Pharma), irinotecan (Accord Healthcare), oxaliplatin (Accord Healthcare), folinic acid (Teva Pharmaceuticals), carboplatin (Teva Pharmaceuticals), cisplatin (Accord Healthcare), docetaxel (Teva Pharmaceuticals), metformin (Accord Healthcare) and gemcitabine (Accord Healthcare). In addition to single-agent drug exposures, commonly used clinical combination regimens based on the above drug panel were evaluated.

ZF larvae were exposed to each chemotherapeutic agent by adding the drug directly to the medium, with a final volume of 15 mL per well containing 12 embryos. Control groups were maintained under identical conditions without drug administration. To ensure consistent and stable drug availability, both the medium and chemotherapeutic solutions were renewed every 24 hours for three consecutive days. This approach was chosen as it represents the most commonly used timeline in ZF xenografting experiments.

**Figure 1:**
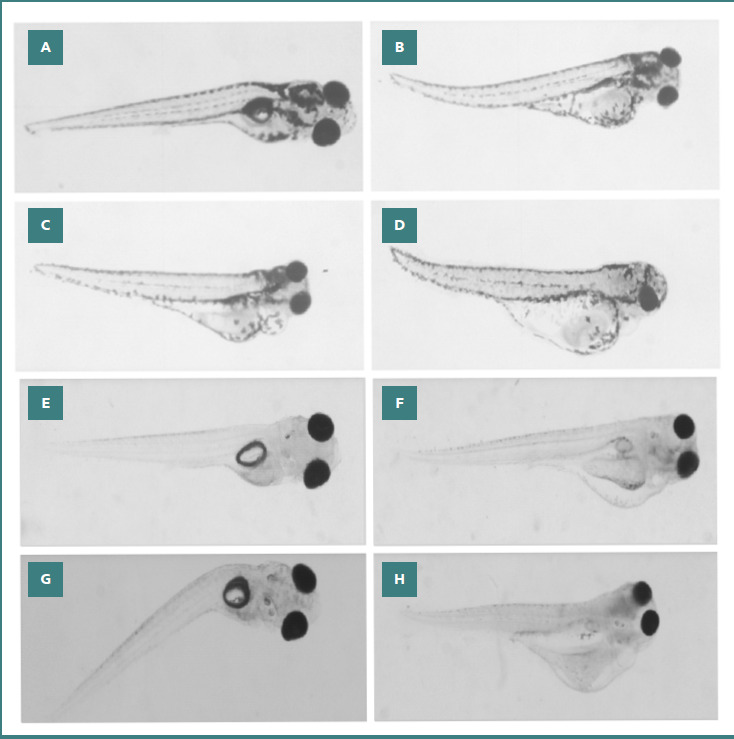
**Representative morphological changes of wild-type and Casper ZF larvae following chemotherapy exposure at Day 3**. Brightfield microscopy images comparing untreated controls to drug-treated larvae. **A–D**, Wild-type Tübingen (TU) strain: **A**, Normal control larva at Day 3, displaying healthy morphology. **B**, Larva treated with cisplatin, exhibiting spinal deformity (curvature) and yolk sac edema. **C**, Larva treated with irinotecan, showing distinct cardiac edema. **D**, Dead larva previously treated with cisplatin, displaying severe toxicity characterized by extensive cardiac and yolk sac edema, and no heartbeat. **E–H**, Transparent Casper strain: **E**, Normal untreated control larva at Day 3. **F**, Larva treated with oxaliplatin, exhibiting yolk sac edema. **G**, Larva treated with irinotecan, exhibiting spinal deformities. **H**, Larva treated with the FOLFIRINOX regimen, presenting with small pericardial edema and yolk sac edema.

Treated ZF larvae were monitored daily for three consecutive days using a Zeiss Stereo Lumar V12 fluorescence stereomicroscope. Images were acquired with a QImaging Retiga 6000 camera. At each time point, mortality and morphological abnormalities, including spinal deformities, pericardial edema, yolk sac edema, or other developmental anomalies, were recorded for all experimental groups. Toxicity evaluation included two parameters: (1) mortality rate, representing the percentage of dead embryos, and (2) AAE, defined as the combined percentage of embryos exhibiting either mortality or any morphological abnormalities (Figure 1A–H).

To ensure reproducibility and to titrate concentrations, three independent toxicity experiments were conducted under identical conditions for the TU strain. Subsequently, to minimize animal usage, the drug concentrations evaluated in the Casper strain were guided by the ranges identified during the primary TU screening. Each experiment using the TU strain included an untreated control group of 12 to 20 larvae, depending on embryo availability. For the Casper strain, untreated control groups consisted of 15 larvae, while all experimental treatment groups contained 12 larvae. All data presented in this study are pooled results from independent experiments.

### Statistical analysis

Descriptive statistics were used to summarize the sample characteristics, with toxicological outcomes reported as absolute frequencies and percentages. Comparative analysis between control and treated groups was performed using Fisher’s exact test for categorical variables. A *P* value < 0.05 was considered statistically significant. All statistical analyses and visualizations were generated using GraphPad Prism version 10 for Windows (GraphPad Software, Boston, Massachusetts, USA). These tests were chosen because we were interested in determining the upper limit of tolerable chemotherapeutic concentration for each agent or regimen at a predetermined time point.

## Results

### Control groups

**Figure 2:**
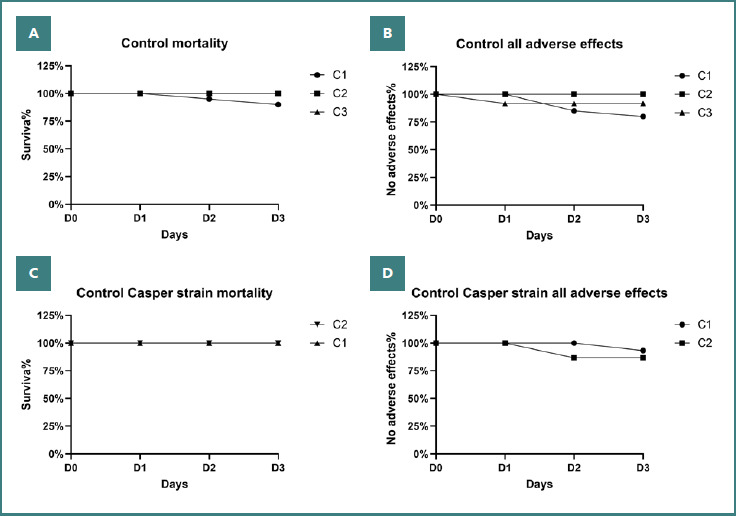
**Control group mortality and all adverse effects (AAE) across independent toxicity assays**. **A–B**, Wild-type TU strain: **A**, Mortality and **B**, AAE across three independent experiments. Control cohorts (C1: n = 20; C2 and C3: n = 12 each) displayed high viability and low baseline AAE over the monitoring period. Survival and AAE remained stable in C2 and C3, with only a mild decline observed in C1. Fisher’s exact test showed no statistically significant differences between TU control groups for either mortality or AAE (Mortality at Day 3: C1 vs C2, P = 0.5161; C1 vs C3, P = 0.5161; C2 vs C3, P = 0.9999. AAE at Day 3: C1 vs C2, P = 0.2709; C1 vs C3, P = 0.6264; C2 vs C3, P = 0.9999). **C–D**, Casper strain: **C**, Mortality, and D, AAE across two independent experiments (C1 and C2). Casper control cohorts exhibited excellent viability, with zero mortality (100% survival) in both C1 and C2. Baseline AAE was minimal, with only 1 AAE recorded in C1 and 2 AAEs recorded in C2. No statistically significant differences in mortality or AAE were observed between the Casper and TU strains at Day 3 (Mortality, P = 0.5113; AAE, P = 0.6812).

Across the three independent toxicity assays, control TU cohorts (C1: *n* = 20; C2 and C3: *n* = 12 each) exhibited high baseline viability throughout the monitoring period. Mortality remained minimal, with no deaths recorded in C2 or C3, whereas two deaths occurred in the C1 cohort during the monitoring period. All adverse effects closely reflected the survival patterns. In C1, two additional larvae exhibited spinal curvature by day 3, while C3 showed a single case of cardiac edema at the same time point; no notable morphological abnormalities were observed in C2. Similarly, the two independent control cohorts for the Casper strain demonstrated excellent viability, with zero mortality observed in either group. Baseline AAEs were also minimal in the Casper cohorts, consisting exclusively of mild spinal curvatures: one instance was recorded in C1, and two instances were observed in C2 (Figure 2A–D).

### Single-agent toxicity profiles

To establish a baseline safety profile and define therapeutic windows for the selected pharmacological agents, we evaluated their individual toxicity in the ZF embryo model. While most chemotherapeutic protocols currently used in a clinical setting use a combination of drugs, we wanted to establish a baseline for each individual compound before moving to multi-agent protocols. For each agent, dose-response relationships were analyzed using cumulative mortality and AAE as primary endpoints. The specific toxicity profiles are detailed below.

**Figure 3:**
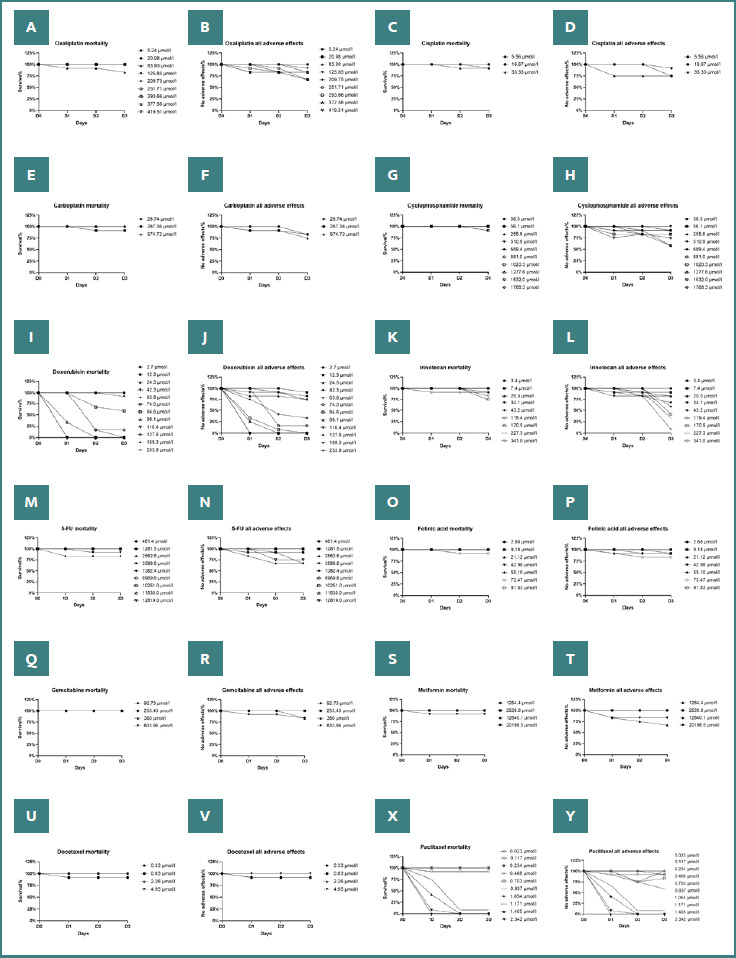
**Survival and toxicity profiles of single chemotherapeutic agents in ZF larvae**. Dose-dependent effects of twelve single agents were monitored over a 3-day exposure period (D0–D3). For each drug, the left panel shows the percentage of survival (mortality), and the right panel shows the percentage of larvae without adverse effects. **A–B**, oxaliplatin; **C–D,** cisplatin; **E–F**, carboplatin; **G–H**, cyclophosphamide; **I–J**, doxorubicin; **K–L,** irinotecan; **M–N**, 5-FU; **O–P**, folinic acid; **Q–R**, gemcitabine; **S–T**, metformin; **U–V**, docetaxel; **X–Y**, paclitaxel. Concentrations are indicated in the legends (µmol/l).

Platinum agents are among the most widely used classes of cytotoxic chemotherapy. Their primary mechanism of action involves forming intra- and inter-strand DNA crosslinks that disrupt replication and transcription, ultimately inducing apoptosis. We evaluated the toxicity profiles of the three commonly used platinum agents: cisplatin, carboplatin, and oxaliplatin across different concentration ranges. Cisplatin was evaluated at three concentrations, resulting in only a slight decline in survival at the highest dose. However, AAE values increased at both the intermediate and highest concentrations, with developmental abnormalities appearing earliest in the highest-dose group. Carboplatin was similarly assessed across three concentration ranges and showed no mortality by day 3, even at the highest concentration tested. Nevertheless, AAE values were greatest in the highest-dose carboplatin group and occurred earlier than in lower concentrations. Oxaliplatin was evaluated across an extended concentration range because initial toxicity assays suggested a pronounced drop in survival and a marked rise in AAE at 83.90 µmol/L. However, upon retesting, this effect was not reproduced, and higher concentrations showed a reasonable toxicity profile, indicating that the initial observation may have been an outlier. Only at substantially higher concentrations did AAE consistently increase. Mortality remained low across all doses tested (Figure 3A–Y).

Cyclophosphamide is an alkylating agent of the nitrogen mustard class that requires hepatic activation to form its cytotoxic metabolites. It is widely used in both solid and hematological malignancies. Cyclophosphamide exposure resulted in a dose-dependent increase in adverse developmental effects, while mortality remained low across all tested concentrations. AAE values stayed near 100% at early time points for concentrations up to ~510 µmol/L but began to decline at higher levels, with mortality remaining minimal. Overall, cyclophosphamide primarily induced morphological toxicity rather than lethality, with pronounced developmental abnormalities emerging at mid- to high micromolar concentrations.

Doxorubicin is an anthracycline chemotherapeutic. Its cytotoxic activity is primarily mediated through DNA intercalation and inhibition of topoisomerase II. Doxorubicin has been tested over a wide range of concentrations, with mortality increasing rapidly in a dose-dependent fashion over 63.8 µmol/L, and AAE reaching pronounced levels above 12.3 µmol/L and 42.3 µmol/L.

**Figure 4:**
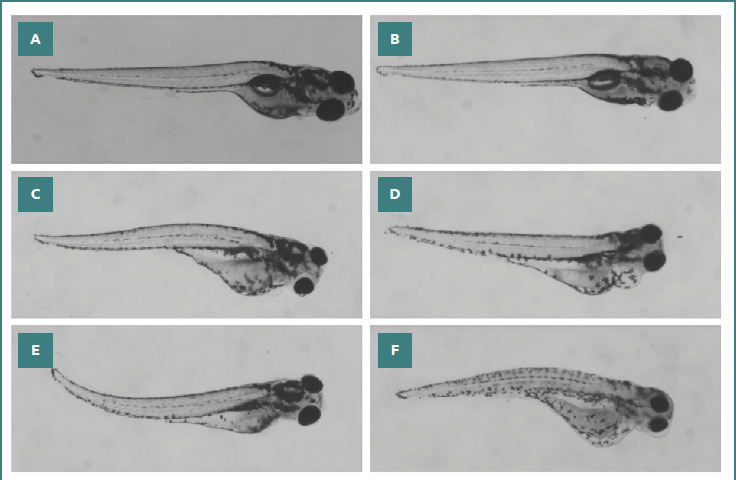
**Representative images of ZF larvae after 72 hours of exposure to varying concentrations of irinotecan**. **A**, Control group: larvae exhibit normal developmental morphology with a straight body axis and clear yolk sac resorption. **B**, Irinotecan 3.4 µmol/L: larvae appear morphologically similar to the control. **C**, Irinotecan 34.1 µmol/L and **D**, 170.5 µmol/L: larvae show initial signs of developmental toxicity, including slight yolk sac edema, axial deviations, and pericardial edema. **E**, Irinotecan 227.3 µmol/L and **F**, 341 µmol/L: at these higher concentrations, significant morphological abnormalities are evident, characterized by pronounced pericardial edema, yolk sac edema, and spinal curvatures. These observations support the finding that irinotecan primarily induces developmental toxicity rather than lethality at moderate to high doses.

Irinotecan is a topoisomerase I inhibitor used in gastrointestinal cancers as part of combination regimens such as 5-FU/folinic acid/irinotecan (FOLFIRI) and 5-FU/folinic acid/irinotecan/oxaliplatin (FOLFIRINOX), as well as in lung, ovarian, and brain tumors. Irinotecan toxicity was dose-dependent, with AAE remaining low at lower and intermediate doses but increasing at higher concentrations, above 170.5 µmol/L. Mortality remained minimal, becoming evident only at the upper range (119.4 µmol/L), indicating that irinotecan primarily caused developmental toxicity at moderate doses (Figure 4A–F).

5-FU is a pyrimidine analog antimetabolite that inhibits thymidylate synthase to prevent DNA synthesis. It is frequently administered in combination with folinic acid (leucovorin) to enhance its cytotoxic effect. 5-FU exposure demonstrated a high safety margin; mortality remained low across all concentrations up to 12819.0 µmol/L, while AAE values indicated only mild developmental toxicity, dipping to approximately 66.7% at mid-range concentrations (3588.6 µmol/L and 7382.4 µmol/L). Folinic acid alone displayed a similarly benign profile consistent with its role as a modulator, with negligible mortality observed up to 91.83 µmol/L. AAE values for folinic acid remained between 83.3% and 100% even at the highest concentrations.

Gemcitabine is a nucleoside metabolic inhibitor that acts as a pyrimidine analog, replacing cytidine during DNA replication. It is the standard of care for pancreatic cancer and is also utilized in bladder, breast, and non-small cell lung cancers. Gemcitabine toxicity was minimal, with mortality remaining absent across all tested concentrations up to 633.95 µmol/L. AAE values remained stable at 100% for lower concentrations but showed a slight, dose-dependent decline at higher levels, dropping to approximately 83.3% at 380 µmol/L and 633.95 µmol/L by Day 3.

Metformin is widely prescribed as the first-line therapy for type 2 diabetes, acting primarily by inhibiting hepatic gluconeogenesis. While not typically utilized as a standard chemotherapeutic, it has demonstrated significant activity in studies as a radiosensitizing agent. Metformin toxicity was generally low. Mortality remained minimal, with survival rates staying at 100% for most concentrations and only a slight decrease to 91.7% observed at the highest tested dose (20198.5 µmol/L). AAE values indicated a dose-dependent increase in developmental toxicity; lower concentrations showed no adverse effects, whereas the two highest concentrations (12640.1 µmol/L and 20198.5 µmol/L) declined to 66.7% by Day 3. Overall, metformin primarily induced morphological toxicity at very high millimolar concentrations, while lethality remained negligible.

Docetaxel and paclitaxel exert their cytotoxic effects by stabilizing microtubules and preventing depolymerization. Despite this shared mechanism, their toxicity profiles in ZF embryos differed significantly. Docetaxel displayed a benign profile across tested concentrations (0.33–4.95 µmol/L), with negligible mortality and AAE values remaining high (91.7–100%). In contrast, paclitaxel exposure resulted in significant, dose-dependent lethality; survival rates dropped rapidly from 100% to 0% at concentrations >1 µmol/L by Day 1, with AAE values similarly plummeting to 0%, indicating severe toxicity and a steep lethal curve.

Following the establishment of concentration ranges in the wild-type TU strain, we conducted comparative experiments with the CSP strain to determine whether this genotype is more susceptible to chemotherapeutic toxicity. Utilizing the initial TU data to guide our concentration selection, we evaluated the single-agent toxicity of 5-fluorouracil, oxaliplatin, irinotecan, and folinic acid.

For 5-FU, the CSP strain demonstrated a high safety margin consistent with the wild-type TU strain. Mortality was minimal, with only one death recorded at 7382.4 µmol/L and one at the highest concentration of 12819.0 µmol/L. The incidence of AAE was similarly low, with two cases at 1281.5 µmol/L, four at 7382.4 µmol/L, and two at 12819.0 µmol/L.

Oxaliplatin exposure yielded comparable safety profiles between the two genetic backgrounds. In the CSP cohorts, survival was excellent; mortality was restricted to a single larva in the 41.95 µmol/L group, with zero deaths at 209.75 µmol/L and 419.51 µmol/L, mirroring the high viability seen in the TU strain. Furthermore, the AAE rates in the Casper strain (two at 41.95 µmol/L, four at 209.75 µmol/L, and three at 419.51 µmol/L) closely tracked with the TU strain.

Folinic acid was highly tolerated in both models. The Casper strain showed 100% survival at both 21.12 µmol/L and 91.83 µmol/L, directly matching the TU strain. AAE rates at Day 3 were also identical across both genotypes, with one adverse event recorded per concentration group.

**Figure 5:**
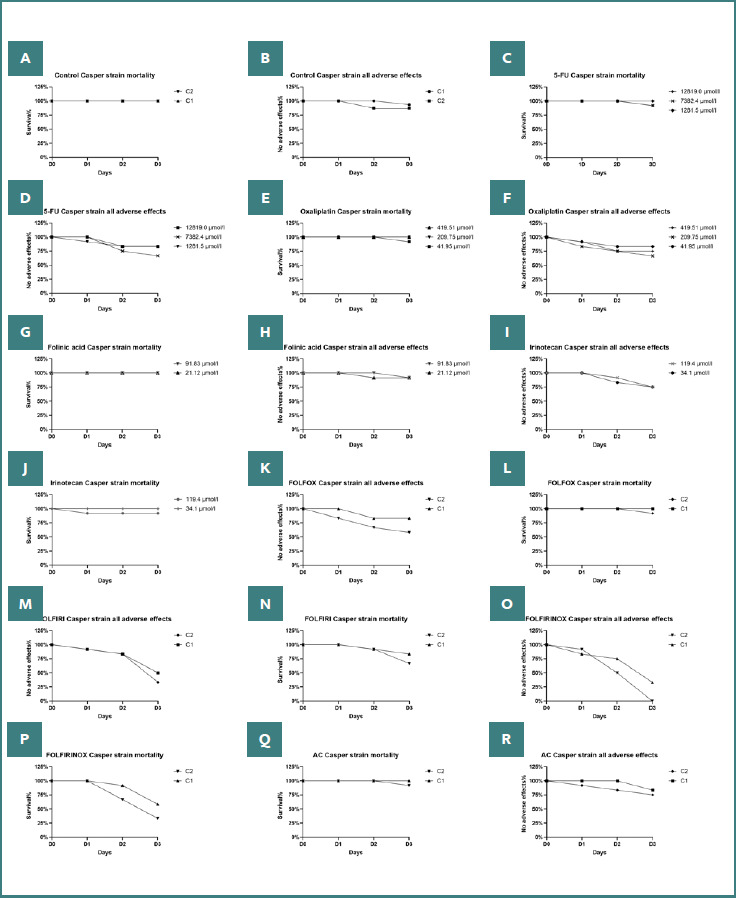
**Survival and toxicity profiles of single agents and combinatorial chemotherapy regimens in Casper strain zebrafish larvae**. Plots illustrating cumulative survival (%) and the percentage of larvae free from adverse effects over a 3-day period (D0–D3). **A–B**, Untreated Controls; C–J, Single-Agent Profiles: Dose-response assessment for **C–D**, 5-Fluorouracil (5-FU); **E–F**, Oxaliplatin; **G–H**, Folinic acid; and **I–J**, Irinotecan at the concentrations indicated in the legends. **K–R**, Multi-Agent Regimens: Comparative toxicity of combination therapies. **K–L**, FOLFOX: Tested at C1 (3588 µmol/L 5-FU + 16.67 µmol/L oxaliplatin + 21 µmol/L folinic acid) and C2 (7328 µmol/L 5-FU + 33.33 µmol/L oxaliplatin + 43 µmol/L folinic acid). **M–N**, FOLFIRI: Tested at C1 (3538 µmol/L 5-FU + 43 µmol/L irinotecan + 21 µmol/L folinic acid) and C2 (7328 µmol/L 5-FU + 114 µmol/L irinotecan + 43 µmol/L folinic acid). **O–P**, OLFIRINOX: Quadruple-agent regimen tested at C1 (3588 µmol/L 5-FU + 43 µmol/L irinotecan + 21 µmol/L folinic acid + 16.67 µmol/L oxaliplatin) and C2 (7328 µmol/L 5-FU + 114 µmol/L irinotecan + 43 µmol/L folinic acid + 33 µmol/L oxaliplatin). **Q–R**, AC Regimen: Doxorubicin and cyclophosphamide combination tested at C1 (1.47 µmol/L doxorubicin + 56 µmol/L cyclophosphamide) and C2 (3.33 µmol/L doxorubicin + 127.5 µmol/L cyclophosphamide).

Irinotecan mortality and AAE were also similar between the two groups. Testing at 34.1 µmol/L and 119.4 µmol/L resulted in identical mortality rates for both the Casper and TU strains: 100% survival at the lower dose and a single recorded death at the higher dose. Morphological evaluations at 119.4 µmol/L revealed three AAE events in the CSP group versus two in the TU group (Figure 5A–R).

### Multi-agent toxicity profiles

After establishing individual safety baselines, we evaluated the toxicity profiles of multi-agent regimens. We examined the standard gastrointestinal regimens (FOLFOX, FOLFIRI, FOLFIRINOX), the AC with or without paclitaxel used in breast cancer, and the combination of 5-FU + metformin.

Evaluation of the three primary gastrointestinal regimens revealed a progressive escalation in both adverse effects and mortality that correlated with increased protocol complexity. FOLFOX demonstrated the most benign profile, with 100% survival across all tested concentrations and minimal developmental toxicity, as AAE values remained at or nearly 100% throughout the observation period. FOLFIRI similarly showed no lethality (100% survival), but induced significant morphological toxicity by Day 3, with AAE values declining to approximately 50–58%. In contrast, the triplet regimen FOLFIRINOX exhibited the most severe toxicity; survival dropped markedly to 58.3% by Day 3 at the highest concentration, accompanied by a profound increase in developmental defects, with AAE values plummeting to 8.3%. This progression highlights cumulative toxicity, in which the addition of irinotecan increases morphological defects, while the full triplet combination significantly impacts both development and survival (Figure 4).

**Figure 6:**
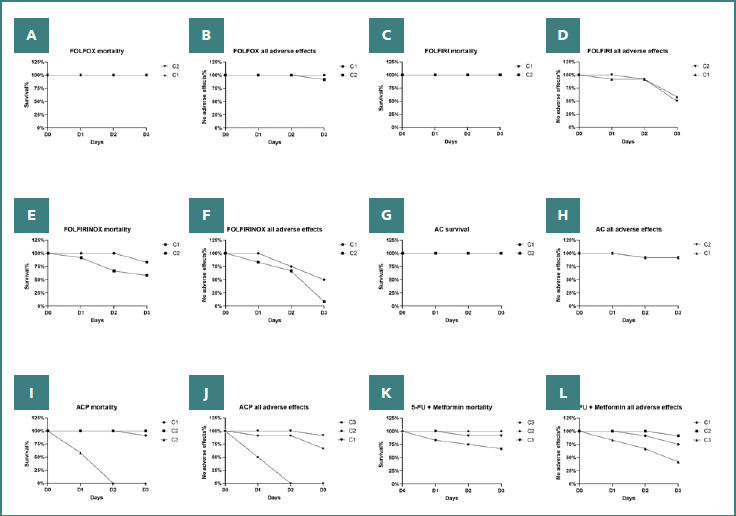
**Survival and toxicity profiles of combinatorial chemotherapy regimens in ZF TU larvae**. Plots illustrating the cumulative survival and AAE for larvae exposed to multi-agent drug combinations over a 3-day period (D0–D3). **A–F**, Standard Colorectal Protocols: **A–B**, FOLFOX: Combinations tested at C1 (3588 µmol/l 5-FU + 16.67 µmol/l oxaliplatin + 21 µmol/l folinic acid) and C2 (7328 µmol/l 5-FU + 33.33 µmol/l oxaliplatin + 43 µmol/l folinic acid). **C–D**, FOLFIRI: Combinations tested at C1 (3538 µmol/l 5-FU + 43 µmol/l irinotecan + 21 µmol/l folinic acid) and C2 (7328 µmol/l 5-FU + 114 µmol/l irinotecan + 43 µmol/l folinic acid). **E–F**, FOLFIRINOX: The quadruple-agent regimen tested at C1 (3588 µmol/l 5-FU + 43 µmol/l irinotecan + 21 µmol/l folinic acid + 16.67 µmol/l oxaliplatin) and C2 (7328 µmol/l 5-FU + 114 µmol/l irinotecan + 43 µmol/l folinic acid + 33 µmol/l oxaliplatin). **G–H**, AC Regime: Combinations of doxorubicin and cyclophosphamide tested at C1 (1.47 µmol/l doxorubicin + 56 µmol/l cyclophosphamide) and C2 (3.33 µmol/l doxorubicin + 127.5 µmol/l cyclophosphamide). **I–J**, ACP Regime: (AC + paclitaxel) tested at C1 (0.94 µmol/l doxorubicin + 38 µmol/l cyclophosphamide + 0.117 µmol/l paclitaxel), C2 (1.46 µmol/l doxorubicin + 56 µmol/l cyclophosphamide + 0.234 µmol/l paclitaxel), and C3 (1.46 µmol/l doxorubicin + 56 µmol/l cyclophosphamide + 0.468 µmol/l paclitaxel). **K–L**, 5-FU + metformin: Combinations tested at C1 (460 µmol/l 5-FU + 1264 µmol/l metformin), C2 (1281 µmol/l 5-FU + 2528 µmol/l metformin), and C3 (3588 µmol/l 5-FU + 12640 µmol/l metformin). Note the significant increase in toxicity metrics consistent with the high drug burden of this intensive regimen.

The AC protocol is widely used in the perioperative setting in breast cancer. Mortality remained absent (100% survival) across both tested concentrations (C1 and C2). Similarly, AAE values indicated only minimal developmental toxicity, remaining at 100% for early time points and stabilizing at 91.7% by Day 3. In contrast, the addition of paclitaxel to the AC regimen resulted in severe, dose-dependent toxicity. While the lowest concentration (C1) maintained high survival and low adverse effects, the highest concentration (C3) caused a rapid decline in viability, with survival dropping to 58.3% by Day 2 and 0% by Day 3. This was mirrored by AAE values, which plummeted to 0% at the highest dose, confirming that the inclusion of the taxane significantly increases the lethal and developmental toxicity of the regimen compared to the AC doublet (Figure 6).

Although standard oncological guidelines do not include the 5-FU and metformin combination, it was evaluated for its potential as a radiosensitizer. At lower and intermediate concentrations (C1 and C2), the regimen maintained a favorable safety profile; mortality was minimal (survival rates of 91.7%-100%), and AAE values remained relatively high (75%–91.7%). However, the highest concentration (C3) induced significant adverse effects. Survival dropped to 66.7% by Day 3, while AAE values fell sharply to 41.7%, suggesting that while the combination is well-tolerated at moderate levels, high-dose exposure triggers both substantial lethality and severe morphological abnormalities (Figure 6A–L).

After establishing baseline toxicity profiles in the wild-type TU strain, we validated these findings in the Casper strain. To ensure a direct, controlled comparison, the CSP cohorts were exposed to the same drug concentrations and regimens as the TU group. This secondary screening was performed to assess whether the specific genotype confers an increased sensitivity to acute chemotherapeutic toxicity.

Strain-dependent toxicity was observed with the FOLFOX regimen. While mortality data were comparable, the Casper genotype exhibited a significantly higher rate of adverse effects (seven total AAEs: two in C1, five in C2) compared to the TU strain (one total AAE). Fisher’s exact test on the pooled datasets confirmed that the Casper strain experienced significantly more AAE at Day 3 (*P* = 0.0479) and no difference in mortality (*P* > 0.9999).

Differences were also observed in the FOLFIRI regimen. In the TU strain, no fatalities were observed at Day 3; in the CSP strain, two deaths were recorded in the C1 group and four in the C2 group; when pooled, the difference was significant (*P* = 0.0011). AAEs were also more prevalent in the CSP subgroups, with six AAE events in C1 and eight AAE events in C2, but there was no statistically significant difference when compared to the TU strain (*P* = 0.5639).

FOLFIRINOX proved to be the most toxic regimen tested. At Day 3, substantial mortality was observed in the Casper strain, with five deaths recorded in the C1 group and eight deaths in the C2 group. Among the survivors, adverse effects were highly prevalent: in C2, virtually all surviving larvae exhibited malformations, while in C1, only four fish retained a normal morphology. However, despite these observed toxicities, when the data were pooled and compared to the TU strain, the differences in mortality (*P* = 0.1425) and AAE (*P* = 0.4936) did not reach statistical significance.

In the AC regimen, no significant differences in the profiles were observed. The slight elevation in mortality (one death in Casper vs. 0 in TU) and AAE frequency (five in Casper vs. two in TU) did not reach statistical significance (Mortality: *P* > 0.9999; AAE: *P* = 0.4158) (Figure 5).

## Discussion

The adoption of zPDX models in personalized oncology and ultimately in the clinic will require the standardization of both the xenografting protocols and the pharmacological profiling. While clinical chemotherapy dosing is rigorously defined by body surface area or weight, ZF dosing remains largely empirical, with significant variability across research groups [9,11]. The primary objective of this study was to establish a robust toxicological baseline for a broad panel of clinically used chemotherapeutics, thereby defining concentration ranges that would not harm ZF embryos. To ensure these experimental ranges were clinically grounded, we compared our dosages to the established peak plasma concentrations observed in human patients (Table 1). Our data demonstrate that the concentrations utilized in the embryos effectively mirror or exceed the logarithmic magnitude of human clinical exposures, particularly those achieved during intravenous bolus administration. Our findings indicate that AAE could serve as a better metric for toxicological profiling than mortality since it captures sublethal developmental toxicity that often precedes death.

**Table 1:** Peak plasma concentration of standard chemotherapeutic agents following conventional intravenous administration

Chemotherapeutic	Administration	Human range	CMax Human	Embryo range tested
Doxorubicin [13,14]	Infusion	30-70 mg/m^2^	1-5 µmol/L	2.7-253.9 µmol/L
5-FU [15,16]	Bolus/Infusion	50-2400 mg/m^2^	100-1000 µmol/L	461-12819 µmol/L
Cyclophosphamide [17]	Infusion	500-1200 mg/m^2^	57-153 µmol/L	38.3-1788.3 µmol/L
Oxaliplatin [18]	Infusion	85-130 mg/m^2^	2.05- 3.05 µmol/L	5.25-419.5 µmol/L
Irinotecan [19]	Infusion	125-350 mg/m^2^	2.8-5.8 µmol/L	3.4-341 µmol/L
Carboplatin [20]	Infusion	75- 450 mg/m^2^	60-128 µmol/L	28.74 – 574.72 µmol/L
Cisplatin [21]	Infusion	50-100 mg/m^2^	15-31 µmol/L	5.56-33.33 µmol/L
Paclitaxel [22]	Infusion	80-175 mg/m^2^	2.5-4.3 µmol/L	0.023-2.342 µmol/L
Docetaxel [23]	Infusion	75-100 mg/m^2^	2.8-3.7 µmol/L	0.33-4.95 µmol/L
Folinic acid [24]	Bolus	50-400 mg/m^2^	10-80 µmol/L	2.68-91.83 µmol/L

Values denote the widely accepted therapeutic peak exposures in adult human oncology patients, converted to micromolar (µmol/L) units to establish a benchmark for the aquatic immersion concentrations used in zebrafish embryos.

Furthermore, ZF embryos in xenografting experiments are subjected to invasive procedures including anesthesia, microinjection, and physical manipulation, which carry a risk of compromising viability or inducing developmental defects. By adhering to non-toxic chemotherapeutic concentrations, we can ensure that any observed adverse effects in the xenograft model are attributable exclusively to the procedural manipulation or the tumor burden itself, rather than to systemic drug toxicity, thereby allowing better standardization of the procedure.

It is important to note that this study relied on only two genotypes, the Casper and the Tübingen wild-type strain. However, genetic background can significantly influence drug sensitivity and metabolic processing. Consequently, significant variations in toxicity profiles are observed in other common lineages [12,25]. Therefore, establishing a specific toxicity baseline for AAE in each strain is essential before xenografting.

Our screening of single agents revealed significant disparities in toxicity profiles, even among drugs with shared mechanisms of action. The most notable difference was observed within the taxane class. Paclitaxel induced severe, dose-dependent lethality and developmental toxicity. This discrepancy could be partially explained by the clinical formulation, which includes Cremophor EL, which is a biologically active polyethoxylated castor oil surfactant known to induce intrinsic toxicity, and ethanol in an approximate 50:50 (v/v) ratio [26]. Even after accounting for the absolute doses of ethanol and Cremophor EL to which the fish were exposed, the concentrations remained within the safety thresholds for ethanol. For example, our 1.4 µmol/L paclitaxel solution contained 0.01% v/v ethanol, well below the 0.1% v/v safety range reported by other groups [12]. Although specific acute toxicity data for Cremophor EL in ZF embryos are unavailable, the manufacturer’s Safety Data Sheet reports an LC50 of approximately 448 mg/L for the surrogate species *Leuciscus idus*. This lethal threshold is roughly four times higher than the 105 mg/L vehicle concentration of our 1,4 µmol/L paclitaxel solution, which resulted in a 100% mortality at 3 days [27]. Consequently, the vehicle alone cannot account for the observed 100% mortality, strongly implicating the intrinsic pharmacological toxicity of paclitaxel. Groups that have used similar formulations of paclitaxel in the clinic have reported similar results, with a safety threshold around 1 µmol/L and a steep drop-off in survival at 2 µmol/L [28]. In contrast, the benign profile of docetaxel may be partly attributed to its reliance on Polysorbate 80 rather than Cremophor EL. This distinction emphasizes the critical importance of testing hospital-grade formulations for each drug, as certain excipients can increase toxicity.

Prodrugs such as cyclophosphamide require hepatic activation (via Cytochrome P450) to exert cytotoxicity. In ZF, the liver primarily develops between 60-72 hpf, allowing metabolic activity to occur [4]. In our model, dose-dependent morphological defects observed with cyclophosphamide confirm that the larval liver is sufficiently functional to generate active toxic metabolites, although this effect was mainly observed at very high levels.

The high safety margins observed with antimetabolites such as 5-FU and gemcitabine are consistent with their specific inhibition of DNA synthesis in dividing tissues. The first 48 hours post-fertilization are defined by organogenesis and rapid cell division. However, after that time point, the embryos transition to the larval stage with functional organ systems by 72 hpf. Mechanistically, these agents act as S-phase-specific inhibitors that disrupt DNA synthesis and repair [29,30]. Because zebrafish tissues significantly decelerate their mitotic rate after the first few days, with most somatic cells transitioning into G1 or G0 phase [31], there are fewer host cells actively undergoing DNA replication to be targeted by these agents. Some groups have tested the effects of 5-FU during the early embryonic period and found that concentrations as low as 10 mg/l (~77 μM) are associated with increased mortality when exposure began at fertilization [32]. By delaying the treatment window, our model effectively bypasses the critical period of organogenesis, thereby significantly reducing the host toxicity of these agents.

Combination regimes are more toxic than individual drugs at comparable concentrations. FOLFOX was well tolerated, likely due to the high safety margins of both 5-FU and oxaliplatin. However, the introduction of irinotecan into the FOLFIRI and FOLFIRINOX regimens led to significant developmental toxicity. The progression to the triplet FOLFIRINOX regimen resulted in a synergistic increase in lethality, mirroring the clinical reality where FOLFIRINOX is associated with higher grades of systemic toxicity in patients compared to doublet therapies. In the AC regime, no significant AAE or mortality was observed, likely because the chemotherapeutic agents were tested at comparatively low concentrations. However, when paclitaxel was added to this regimen, it significantly accelerated mortality and caused it to appear at lower doses than those observed in the single-agent paclitaxel group.

Although the Casper strain displayed single-agent tolerance comparable to the wild-type TU strain, it proved significantly more susceptible to the synergistic toxicity of combination therapies. We observed a marked increase in adverse effects with the FOLFOX regimen and elevated mortality with the FOLFIRI regimen when compared to the TU strain. Because of this, establishing strain-specific toxicity thresholds is essential to avoid confounding drug-induced systemic toxicity with xenograft-related mortality.

## Conclusion

This study provides a comprehensive toxicity framework for 11 clinically relevant chemotherapeutic agents and their combination regimens in the ZF embryo model. We suggest that AAE serves as a more sensitive and predictive metric than lethality for defining the therapeutic window, capturing essential sublethal developmental toxicity. Furthermore, it is crucial to test the precise drug formulation available, as certain excipients may interfere with the model and affect the results. Our comparative profiling of the Casper strain revealed that genetic background influences tolerance to combinatorial regimens, while single-agent toxicity profiles were largely consistent between strains. It is also necessary to test multi-agent protocols, as our findings demonstrate that toxicity can be observed significantly lower than when single agents are used. Therefore, strain-specific testing is recommended to ensure accurate toxicity assessment. These findings aim to advance the standardization of ZF xenografting, supporting its potential translation to clinical applications.

## Data Availability

Further data is available from the corresponding author upon reasonable request.

## References

[1] Mukherjee S, Mohanty AK, Chinnadurai RK, Barman DD, Poddar A (2024). Zebrafish: A Cost-Effective Model for Enhanced Forensic Toxicology Capabilities in Low-and Middle-Income Countries. Cureus.

[2] Strähle U, Scholz S, Geisler R, Greiner P, Hollert H, Rastegar S (2012). Zebrafish embryos as an alternative to animal experiments--a commentary on the definition of the onset of protected life stages in animal welfare regulations. Reprod Toxicol.

[3] Howe K, Clark MD, Torroja CF, Torrance J, Berthelot C, Muffato M (2013). The zebrafish reference genome sequence and its relationship to the human genome. Nature.

[4] Nawaji T, Yamashita N, Umeda H, Zhang S, Mizoguchi N, Seki M (2020). Cytochrome P450 Expression and Chemical Metabolic Activity before Full Liver Development in Zebrafish. Pharmaceuticals (Basel).

[5] Keij FM, Koch BEV, Lozano Vigario F, Simons SHP, van Hasselt JGC, Taal HR (2021). Zebrafish larvae as experimental model to expedite the search for new biomarkers and treatments for neonatal sepsis. J Clin Transl Sci.

[6] Zebrafish Lines at ZIRC [Internet] https://zebrafish.org/fish/lineAll.php.

[7] Di Franco G, Usai A, Piccardi M, Cateni P, Palmeri M, Pollina LE (2022). Zebrafish patient-derived xenograft model to predict treatment outcomes of colorectal cancer patients. Biomedicines.

[8] Mendes RV, Ribeiro JM, Gouveia H, Rebelo de Almeida C, Castillo-Martin M, Brito MJ (2025). Zebrafish Avatar testing preclinical study predicts chemotherapy response in breast cancer. NPJ Precis Oncol.

[9] Song F, Yi X, Zheng X, Zhang Z, Zhao L, Shen Y (2025). Zebrafish patient-derived xenograft system for predicting carboplatin resistance and metastasis of ovarian cancer. Drug Resist Updat.

[10] Hua X, Wu X, Xu K, Zhan P, Liu H, Zhang F (2023). Zebrafish patient-derived xenografts accurately and quickly reproduce treatment outcomes in non-small cell lung cancer patients. Exp Biol Med (Maywood).

[11] Lindahl G, Fjellander S, Selvaraj K, Vildeval M, Ali Z, Almter R (2024). Zebrafish tumour xenograft models: a prognostic approach to epithelial ovarian cancer. NPJ Precis Oncol.

[12] Loucks E, Carvan MJ (2004). Strain-dependent effects of developmental ethanol exposure in zebrafish. Neurotoxicol Teratol.

[13] Speth PA, van Hoesel QG, Haanen C (1988). Clinical pharmacokinetics of doxorubicin. Clin Pharmacokinet.

[14] Barpe DR, Rosa DD, Froehlich PE (2010). Pharmacokinetic evaluation of doxorubicin plasma levels in normal and overweight patients with breast cancer and simulation of dose adjustment by different indexes of body mass. Eur J Pharm Sci.

[15] Casale F, Canaparo R, Serpe L, Muntoni E, Pepa CD, Costa M (2004). Plasma concentrations of 5-fluorouracil and its metabolites in colon cancer patients. Pharmacol Res.

[16] Diasio RB, Harris BE (1989). Clinical pharmacology of 5-fluorouracil. Clin Pharmacokinet.

[17] Moore MJ (1991). Clinical pharmacokinetics of cyclophosphamide. Clin Pharmacokinet.

[18] U.S. Food and Drug Administration (2020). ELOXATIN (oxaliplatin injection) for intravenous use: prescribing information [Internet]. Silver Spring (MD): FDA.

[19] Pfizer (2022). CAMPTOSAR® (irinotecan hydrochloride) injection for intravenous use: prescribing information [Internet]. https://labeling.pfizer.com/ShowLabeling.aspx?id=533.

[20] Oguri S, Sakakibara T, Mase H, Shimizu T, Ishikawa K, Kimura K (1988). Clinical pharmacokinetics of carboplatin. J Clin Pharmacol.

[21] Urien S, Brain E, Bugat R, Pivot X, Lochon I, Van ML (2005). Pharmacokinetics of platinum after oral or intravenous cisplatin: a phase 1 study in 32 adult patients. Cancer Chemother Pharmacol.

[22] U.S. Food and Drug Administration (2011). TAXOL® (paclitaxel) injection: prescribing information, patient information included [Internet]. Silver Spring (MD): FDA.

[23] Kenmotsu H, Tanigawara Y (2015). Pharmacokinetics, dynamics and toxicity of docetaxel: Why the Japanese dose differs from the Western dose. Cancer Sci.

[24] Anderson JH, Kerr DJ, Setanoians A, Cooke TG, McArdle CS (1992). A pharmacokinetic comparison of intravenous versus intra-arterial folinic acid. Br J Cancer.

[25] Padovani BN, Abrantes do Amaral M, Fénero CM, Paredes LC, Boturra de Barros GJ, Xavier IK (2022). Different wild type strains of zebrafish show divergent susceptibility to TNBS-induced intestinal inflammation displaying distinct immune cell profiles. Curr Res Immunol.

[26] Gelderblom H, Verweij J, Nooter K, Sparreboom A (2001). Cremophor EL: the drawbacks and advantages of vehicle selection for drug formulation. Eur J Cancer.

[27] BASF SE (2025). Kolliphor® EL: safety data sheet. [Internet]. Ludwigshafen (Germany): BASF SE.

[28] D’Iglio C, Famulari S, Capparucci F, Gervasi C, Cuzzocrea S, Spanò N (2023). Toxic Effects of Gemcitabine and Paclitaxel Combination: Chemotherapy Drugs Exposure in Zebrafish. Toxics.

[29] Plunkett W, Huang P, Gandhi V (1995). Preclinical characteristics of gemcitabine. Anticancer Drugs.

[30] Zhang N, Yin Y, Xu SJ, Chen WS (2008). 5-Fluorouracil: mechanisms of resistance and reversal strategies. Molecules.

[31] Sugiyama M, Sakaue-Sawano A, Iimura T, Fukami K, Kitaguchi T, Kawakami K (2009). Illuminating cell-cycle progression in the developing zebrafish embryo. Proc Natl Acad Sci U S A.

[32] Kovács R, Bakos K, Urbányi B, Kövesi J, Gazsi G, Csepeli A (2016). Acute and sub-chronic toxicity of four cytostatic drugs in zebrafish. Environ Sci Pollut Res Int.

